# Identification of a hypoxia-related gene prognostic signature in colorectal cancer based on bulk and single-cell RNA-seq

**DOI:** 10.1038/s41598-023-29718-2

**Published:** 2023-02-13

**Authors:** Yihuan Qiao, Xunliang Jiang, Yaoting Li, Ke Wang, Rujie Chen, Jun Liu, Yongtao Du, Li Sun, Jipeng Li

**Affiliations:** 1 Department of Digestive Surgery, Honghui Hospital, Xi’an Jiaotong University, Xi’an, 710054 Shaanxi China; 2 State Key Laboratory of Cancer Biology, Department of Biochemistry and Molecular Biology, Air Force Medical University, Xi’an, 710032 Shaanxi China; 3 Department of Gastrointestinal Surgery, The First Affiliated Hospital of Air Force Medical University, Xi’an, 710032 Shaanxi China; 4 Xi’an Gaoxin No. 1 High School, Xi’an, 710119 Shaanxi China

**Keywords:** Cancer, Cancer genetics

## Abstract

Colorectal cancer (CRC) is the most common and fatal tumor in the gastrointestinal system. Its incidence and mortality rate have increased in recent years. Hypoxia, a persistent physiological tumor feature, plays a vital role in CRC tumorigenesis, metastasis, and tumor microenvironment (TME). Therefore, we constructed a hypoxia-related gene (HRG) nomogram to predict overall survival (OS) and explored the role of HRGs in the CRC TME. The Cancer Genome Atlas (TCGA) dataset was used as the training set, and two Gene Expression Omnibus datasets (GSE39582 and GSE103479) were used as the testing sets. HRGs were identified using the Gene Set Enrichment Analysis (GSEA) database. An HRG prognostic model was constructed in the training set using the least absolute shrinkage and selection operator regression algorithm and validated in the testing sets. Then, we analyzed tumor-infiltrating cells (TICs) using the cell-type identification by estimating relative subsets of RNA transcripts (CIBERSORT) algorithm. Furthermore, single-cell next-generation RNA sequencing (RNA-seq) was used to investigate HRG expression in different TICs in the GSE139555 dataset. Finally, reverse transcription polymerase chain reactions (RT-PCR) were used to validate HRG mRNA expression in ten pairs of CRC normal and cancer tissue samples. A six HRG prognostic signature was constructed, with a superior OS prediction ability in CRC patients (area under the receiver operating characteristic curve (AUC) at one year: 0.693, AUC at three years: 0.712, and AUC at five years: 0.780). GSEA enrichment analysis identified six pathways enriched in the high-risk group. The TIC analysis indicated that the high-risk group had lower T-cell expression and higher neutrophil expression than the low-risk group. Furthermore, immune-related genes had an inseparable relationship with the HRG prognostic signature. Based on single-cell RNA-seq data, we found elevated hexokinase 1 (*HK1*) and glucose-6-phosphate isomerase (*GPI*) gene expression in natural killer (NK) and CD8^+^ T cells. RT-PCR in ten CRC normal-tumor tissue pairs showed that expression of the signature’s six HRGs varied differently in cancerous and paracancerous tissues. The constructed HRG signature successfully predicted the OS of whole-stage CRC patients. In addition, we showed that the signature’s six HRGs were closely associated with the TME in CRC, where hypoxia inhibits the antitumor function of T cells.

## Introduction

Colorectal cancer (CRC) is the most commonly diagnosed gastrointestinal tumor, with the third highest incidence and second highest mortality^1^. CRC includes colon adenocarcinoma (COAD) and rectal adenocarcinoma. Due to delayed diagnosis, rapid progression, and early metastasis, CRC patients’ overall survival (OS) is far from clinically satisfactory, even with recent dramatic advances in CRC treatment strategies^2,3^. Therefore, a superior prediction signature is urgently needed to ameliorate the current CRC diagnosis and treatment situation.

An important feature of solid tumors is hypoxia (low oxygen levels), which contributes to poor prognosis and therapeutic outcomes. Numerous studies have shown that hypoxia plays a crucial role in various critical cancer aspects, including angiogenesis^4^, genome instability^5^, metabolic reprogramming^6^, epithelial-mesenchymal transition (EMT)^7^, immune evasion^8^, and therapy resistance^9^. Hypoxia induces increased expression of related genes and the creation of intratumoral oxygen gradients, such as reactive oxygen species (ROS), resulting in tumor plasticity and heterogeneity and enhanced tumor invasion and metastasis^10^.

Hypoxia drives cancer progression mainly through hypoxia-inducible factors (HIFs). During normoxia, HIF-1α is hydroxylated and binds to the von Hippel-Lindau tumor suppressor (VHL) for ubiquitination^11^. HIF-1α rapidly accumulates in cells and drives gene transactivation during hypoxia. HIF-1α facilitates the glycolytic process by transactivating the expression of key glycolysis signaling pathway enzymes, including hexokinase 2 (HK2) and pyruvate kinase muscle isozyme M2 (PKM2)^12^. While HIF-1α is highly sensitive to oxygen concentration, HIF-1β is constitutively expressed regardless of the oxygen concentration^11^. The HIF-1α/HIF-1β complex can translocate to the nucleus and modulate genes with hypoxia-response consensus sequences^13^. In addition, HIF-1 inhibition by meloxicam had an anticancerous effect on hepatocellular carcinoma (HCC), contributing to caspase-dependent apoptosis of HCC cells during hypoxia^14^. In contrast to universal HIF-1α expression in almost all cells, HIF-2α is selectively expressed in tumor stem and endothelial cells. However, the underlying direct or indirect interaction mechanisms between hypoxia and CRC remain unclear.

**Figure 1:**
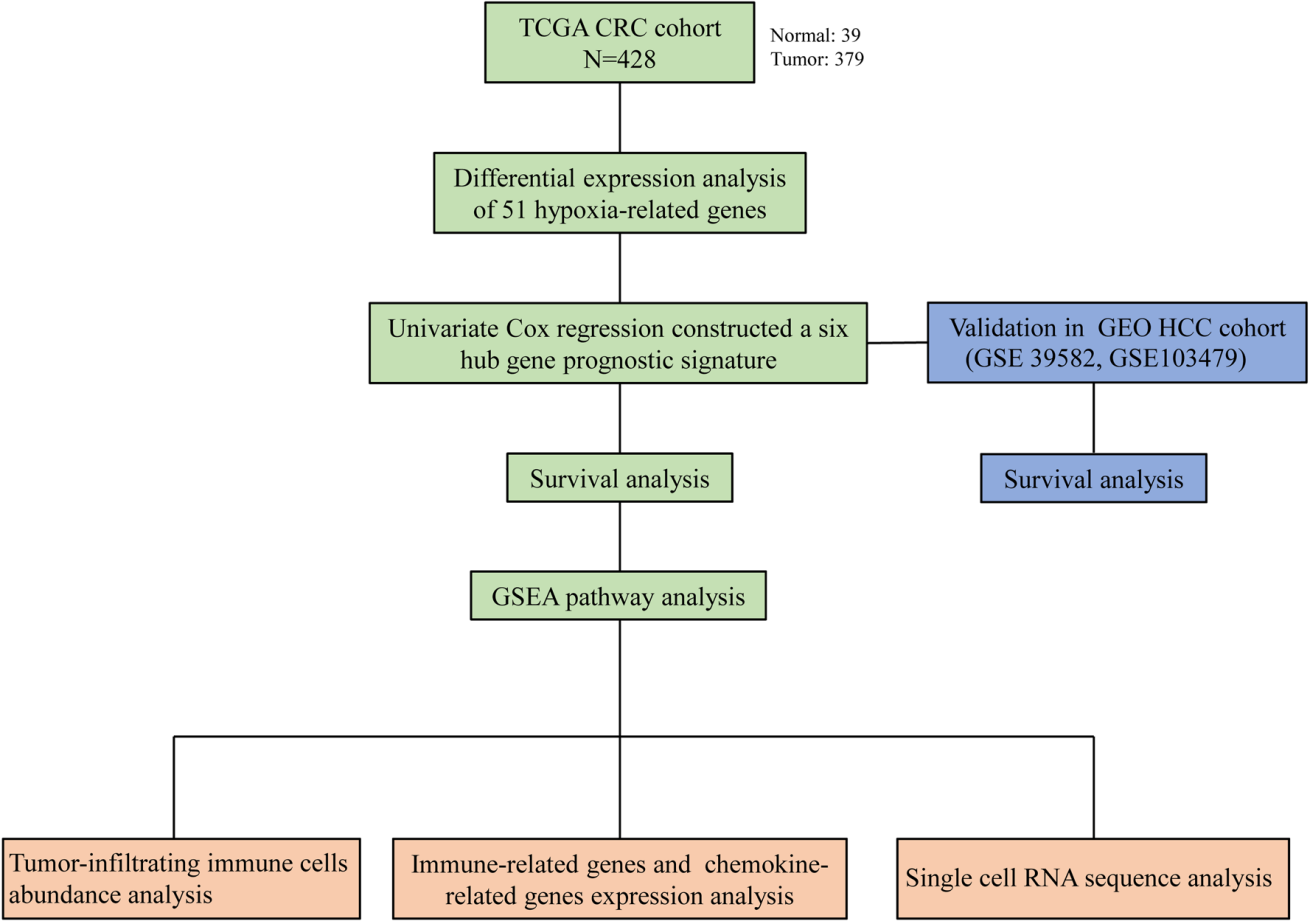
The study workflow.

Here, we evaluated the interaction between hypoxia-related genes (HRGs) and CRC, which could provide prognostic information for CRC patients. We developed a novel hypoxia gene signature independent of the current clinicopathologic characteristics and staging system through a series of systematic analyses to improve CRC prognostication.The study workflow is depicted in Fig. 1.

## Materials and methods

### Data acquisition

CRC patients’ gene expression and corresponding clinicopathological features were obtained for the CRC cohort in The Cancer Genome Atlas (TCGA) and the Gene Expression Omnibus (GEO) datasets GSE39582 and GSE103479. A single-cell next-generation RNA sequencing (RNA-seq) dataset for CRC patients was also obtained from the GEO database (GSE139555)^15^.The datasets generated and/or analysed during the current study are available in the TCGA and GEO repository, https://portal.gdc.cancer.gov/, https://www.ncbi.nlm.nih.gov/geo/.

### Identification of differentially expressed and prognostic genes

Fifty-one HRGs were extracted from Gene Set Enrichment Analysis (GSEA) database^16^. The linear models for microarray data (*LIMMA*) R package was used to identify differentially expressed genes (DEGs) between paracancerous and cancerous samples based on a *P* < 0.05 and |log_2_ fold change|> 2. OS-related DEGs were identified using a univariable Cox regression^17^.

### HRG prognostic signature construction

The most predictive signature was constructed using multivariate Cox regression analyses with the *survival* R package. CRC patients were divided into two groups based on their median risk scores: high-risk and low-risk. Receiver operating characteristic (ROC) curves and Kaplan–Meier (K–M) curves were used to evaluate the risk model’s predictive capability^18^. In addition, univariate and multivariate Cox regression analyses were used to explore the risk score model’s prognostic efficiency independent of other clinicopathological features^17^.

### Functional enrichment analysis

GSEA was performed using the GSEA software (version 4.0.1) with 1000 permutation numbers. The cut-off criteria were gene size ≥ 15, |normalized enrichment score (NES)|> 1.5, and nominal *P* < 0.05.

### Tumor-infiltrating cell analysis

The abundances of 22 tumor-infiltrating cell (TIC) types were estimated using the cell type identification by estimating relative subsets of RNA transcripts (CIBERSORT) algorithm^19^ in the TCGA cohort, identifying significant results based on a *P* < 0.05.

### Immune-related gene analysis

We analyzed the expression of 55 immune-related genes and nine chemokine-related genes extracted from the GSEA database to further explore the potential relationship between risk score and tumor immunity.

### Gene expression quantification

Ten paired CRC tumor-normal tissues were obtained from patients in the Xijing Hospital (Xian, China). Informed consent was taken from all the patients as previously described^20^. Messenger RNA (mRNA) was extracted from each tissue using the TRIzol method (Invitrogen, Carlsbad, CA, USA). Quantitative real-time reverse transcription polymerase chain reactions (RT-PCR) were performed using SYBR-green PCR MasterMix (TaKaRa, Tokyo, Japan). Information related to the RT-PCR is listed in Table S1.

### Single-cell RNA-seq and statistical analyses

The single-cell RNA-seq analysis was performed using the Tumor Immune Single-cell Hub 2 web service (http://tisch.comp-genomics.org/home/)^21^ using the uniform manifold approximation and projection (UMAP) method to reduce dimensionality and visualize the clustering results^21^. The mRNA expression of different cells was also visualized using UMAP distribution figures. The R statistical software (version 3.63; http://www.r-project.org/) was used to perform statistical analysis. All statistical tests were two-sided, and a *P* < 0.05 was considered statistically significant.

## Results

### HRG prognostic signature construction

**Figure 2:**
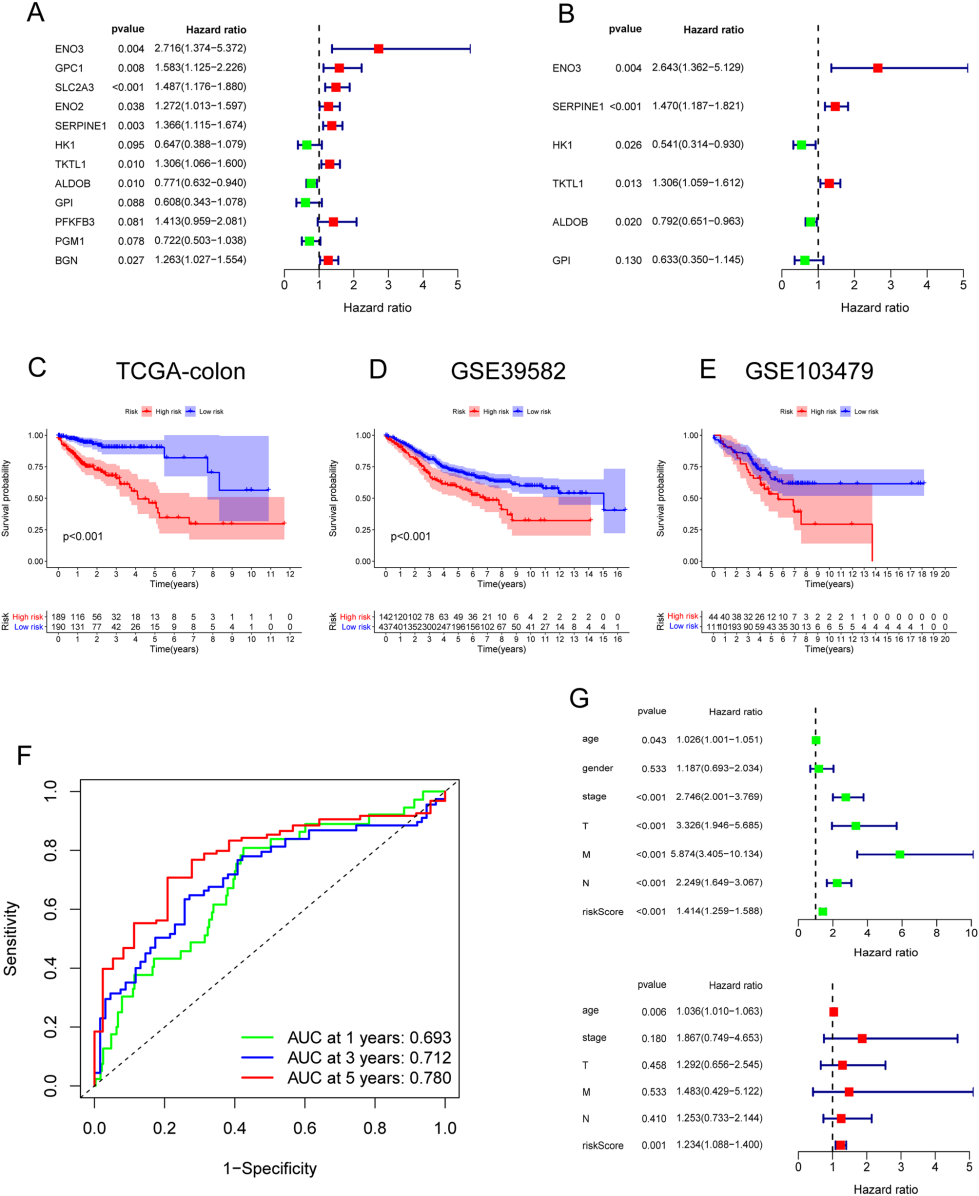
Construction of the prognostic HRG model. (**A**) Differentially expressed HRGs in CRC patients and healthy individuals in the TCGA. (**B**) Multivariate and univariate Cox regression analyses of the prognostic model’s six HRGs. (**C**) K–M curves of OS in the TCGA dataset. (**D**) K–M curves of OS in the GEO dataset. (**E**) ROC curve for the TCGA dataset. (**F**) Multivariate and univariate Cox regression analyses of risk scores and clinicopathological characters.

Using the univariate Cox regression analysis, we identified 12 prognostic hypoxia-related DEGs in the TCGA colon cancer database (Fig. 2A). A superior signature containing six prognostic HRGs was constructed based on multivariate Cox regression analyses of the TCGA (training) and GEO (testing) sets. Three protective genes were significantly associated with prognosis (Fig. 2B): hexokinase 1 (*HK1*; hazard ratio [HR] = 0.541, 95% confidence interval [CI] 0.314–0.930), aldolase B (*ALDOB*; HR = 0.792, 95% CI 0.651–0.963), and glucose-6-phosphate isomerase (*GPI*; HR = 0.633, 95% CI 0.350–1.145). In addition, three hazard genes were identified: enolase 3 (*ENO3*; HR = 2.643, 95%CI 1.362–5.129), serpin family E member 1 (*SERPINE1*; HR = 1.470, 95% CI 1.187–1.821), and transketolase-like protein 1 (*TKTL1*; HR = 1.306, 95% CI 1.059–1.621). The coefficients were also verified by multivariate Cox analysis (derived using the forward–backward method; Table 1). The CRC patients’ risk scores were calculated as follows: (0.971881 × expression [*ENO3*]) + (0.385271 × expression [*SERPINE1*]) + (0.267247 × expression [*TKTL1*]) − (0.61523 × expression [*HK1*]) − (0.23354 × expression [*ALDOB*]) − (0.45796 × expression [*GPI*]).

**Table 1:** Coefficients of hypoxia-related genes by multivariate Cox analysis.

Hypoxia-related genes	Coefficients	HR	HR.95L	HR.95H	*P* value
ENO3	0.971881	2.642911	1.361901	5.128847	0.004065
SERPINE1	0.385271	1.470013	1.186897	1.820661	0.000416
HK1	− 0.61523	0.540516	0.314043	0.93031	0.026371
TKTL1	0.267247	1.306363	1.058941	1.611596	0.012613
ALDOB	− 0.23354	0.791726	0.65066	0.963376	0.019667
GPI	− 0.45796	0.632574	0.34951	1.144889	0.130292

### Prognostic HRG signature validation

We evaluated each COAD case’s risk score and divided patients into two risk groups (low and high) according to the median risk score. K–M curves indicated that the high-risk group had a poorer OS rate than the low-risk group in both training (TCGA cohort; Fig. 2C) and testing (GSE39582; Fig. 2D) sets. In addition, another CRC cohort (GSE103479) was used to further validate the HRG signature’s prognostic value, with the high-risk group showing worse prognoses than the low-risk group (Fig. 2E). The HRG signature’s area under the ROC curve (AUC) indicated good predictability at one (AUC = 0.693), three (AUC = 0.712), and five (AUC = 0.780) years (Fig. 2F). Then, univariate and multivariate Cox regression analyses were used to estimate whether the risk model could be an independent clinically prognostic factor. The results showed that age, sex, American Joint Committee on Cancer (AJCC) stage, tumor (T) stage, node (N) stage, and metastasis (M) stage were significantly correlated with the risk score (Fig. 2G).

**Figure 3:**
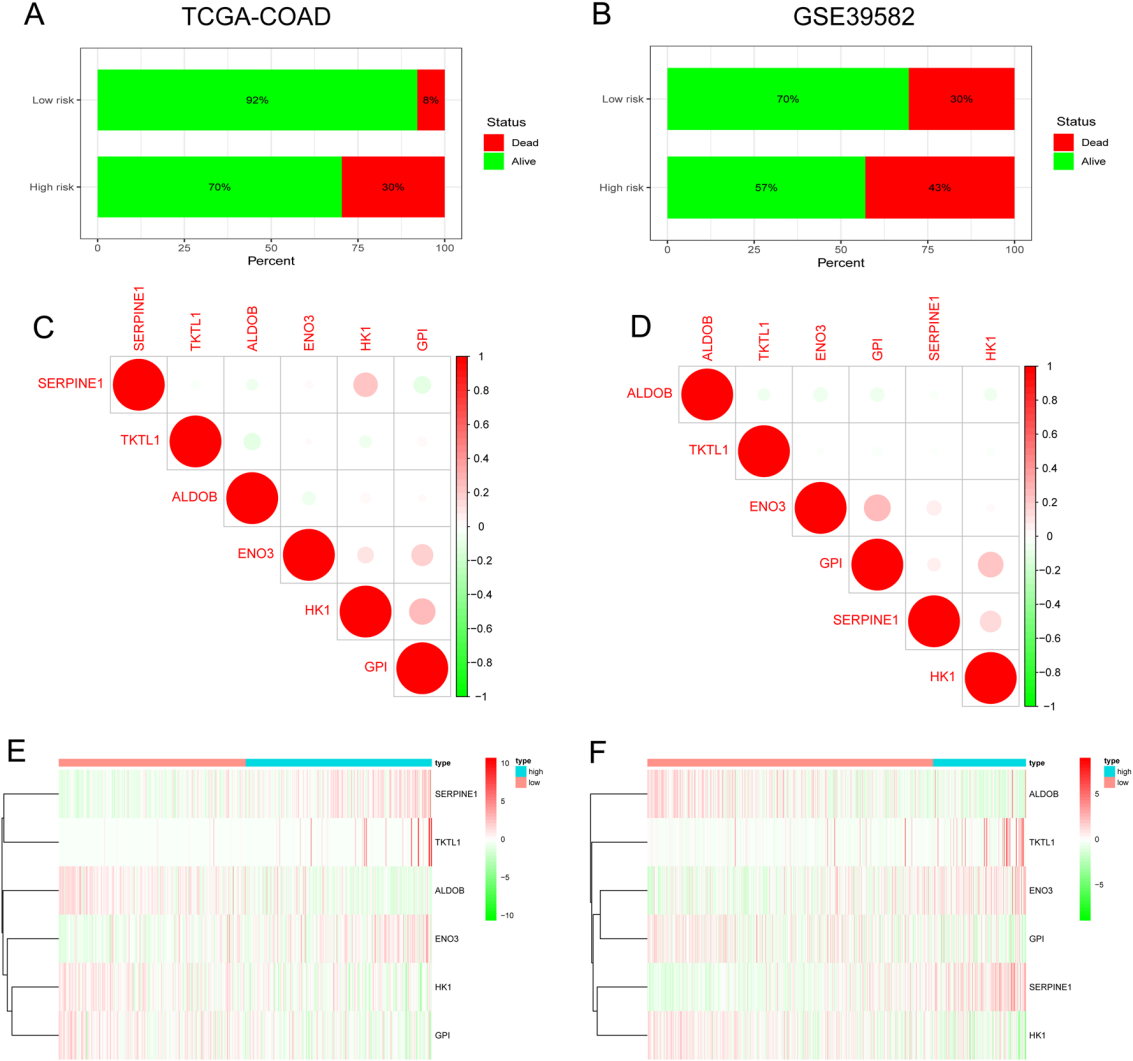
Prognostic HRG model verification. Percentage survival status for the low- and high-risk groups in the (**A**) TCGA and (**B**) GEO datasets. Correlation analyses of the signature’s six HRGs in the (**C**) TCGA and (**D**) GEO datasets. (**E**, **F**) Heatmaps of the six HRGs in the GEO dataset.

Furthermore, the high-risk group contained a higher percentage of cases with death outcomes than the low-risk group in both TCGA (high risk: 30% with dead status, 70% with alive status; low risk: 8% with dead status, 92% with alive status) and GEO (high risk: 43% with dead status, 53% with alive status; low risk: 30% with dead status, 70% with alive status) datasets (Fig. 3A,B). Using correlation analyses, we found that the signature’s six HRGs were independent in both databases (Fig. 3C,D). Moreover, the expression of key genes in both datasets was plotted in the heatmap (Fig. 3E,F).

### Functional annotation

**Figure 4:**
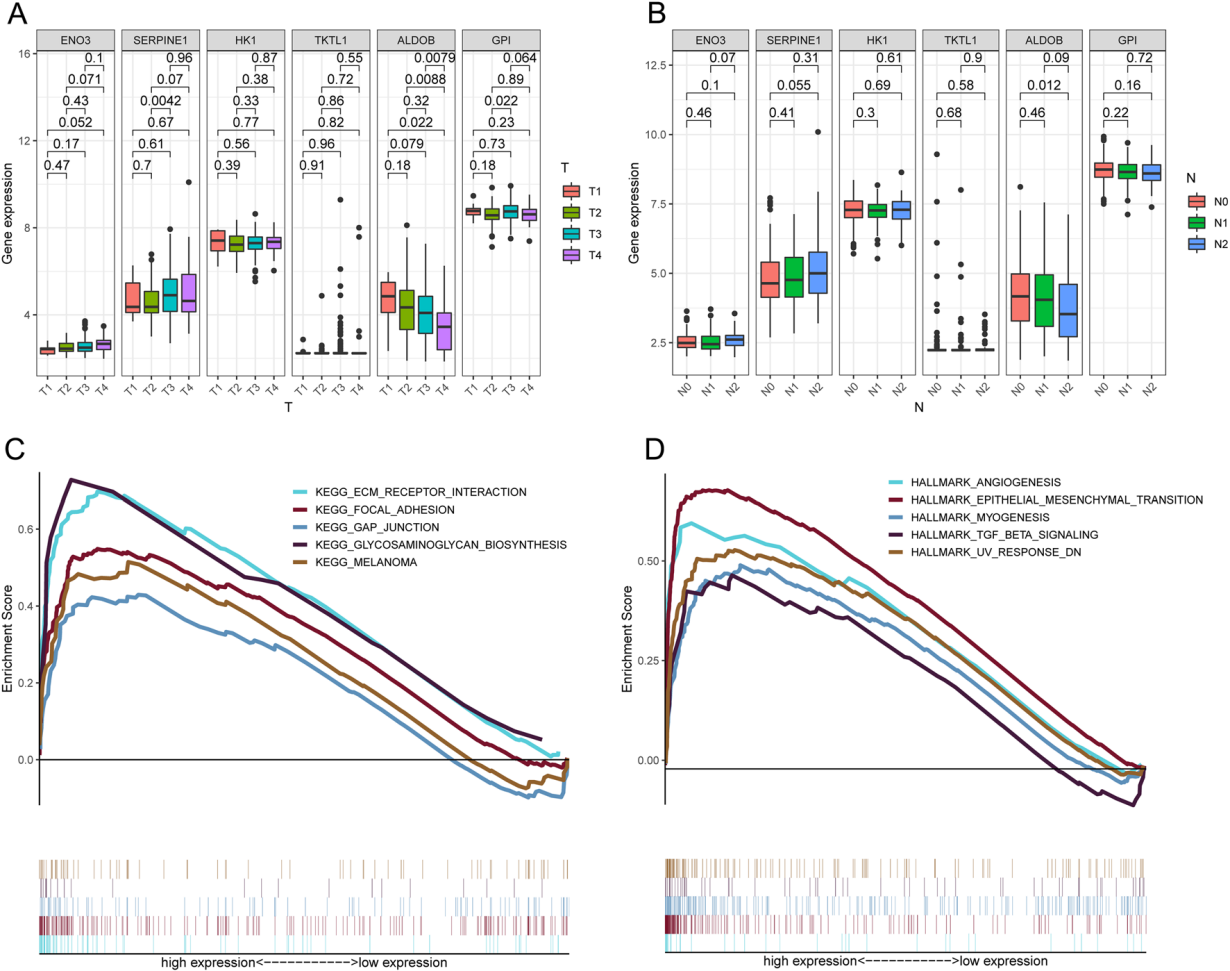
Function enrichment. Differential expression of the signature’s six HRGs in (**A**) T and (**B**) N stage tumors. (**C**, **D**) GSEA enrichment analyses.

In addition, we analyzed the expression of the signature’s six HRGs in different tumor stages. *ALDOB* expression was significantly lower at T4 and N2 (Fig. 4A,B). Using GSEA enrichment analysis, we identified six pathways enriched in the high-risk group, including extracellular matrix receptor interaction, focal adhesion, gap junction, glycosaminoglycan biosynthesis, and melanoma pathways. In addition, five cancer hallmarks were enriched in the high-risk group: angiogenesis, EMT, myogenesis, transforming growth factor (TGF)-β signaling, and UV response DNA pathways (Fig. 4C,D).

### Immune correlation analysis

**Figure 5:**
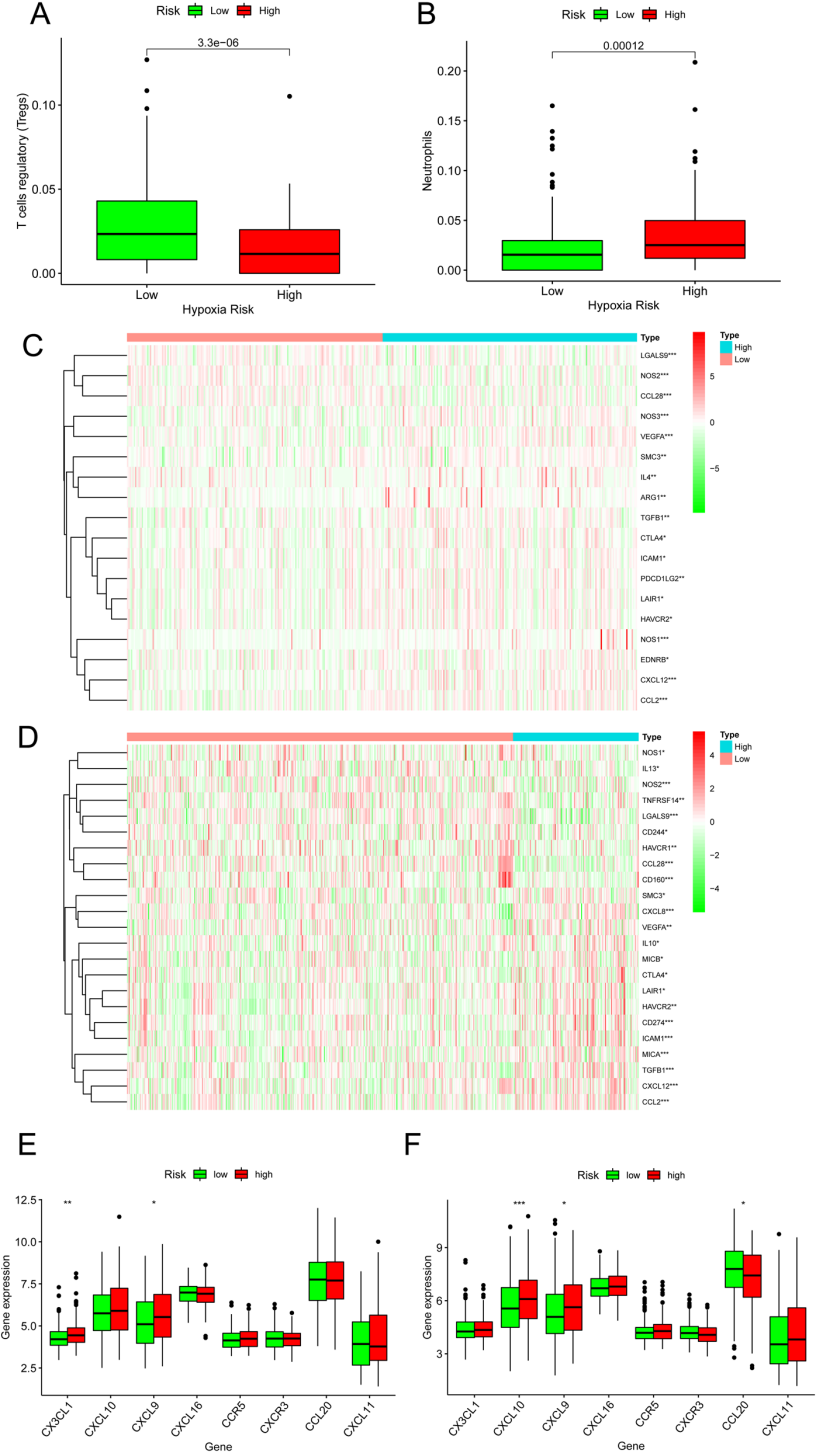
TICs and immune genes analysis. Expression of (**A**) regulatory T cells and (**B**) neutrophils in the TCGA dataset. (**C**, **D**) Heatmaps of immune-related gene expression. Chemokine expression in the (**E**) TCGA and (**F**) GSE39582 datasets.

The expression of 16 TICs in the TCGA database indicated that the high-risk group had lower regulatory T cell expression and higher neutrophil expression than the low-risk group (Fig. 5A,B). Moreover, 18 immune-related genes were closely associated with risk scores in the TCGA dataset (Fig. 5C) and 23 in the GEO dataset (Fig. 5D). Additionally, we explore the underlying relationship between risk score and chemokine expression in both datasets. *CXCL9* expression was significantly increased in the high-risk group in both databases. In contrast, the high-risk group had higher *CX3CL1* expression in the TCGA dataset but higher *CXCL10* expression in the GEO dataset (Fig. 5E,F).

### Single-cell RNA-seq analysis of HRGs

**Figure 6:**
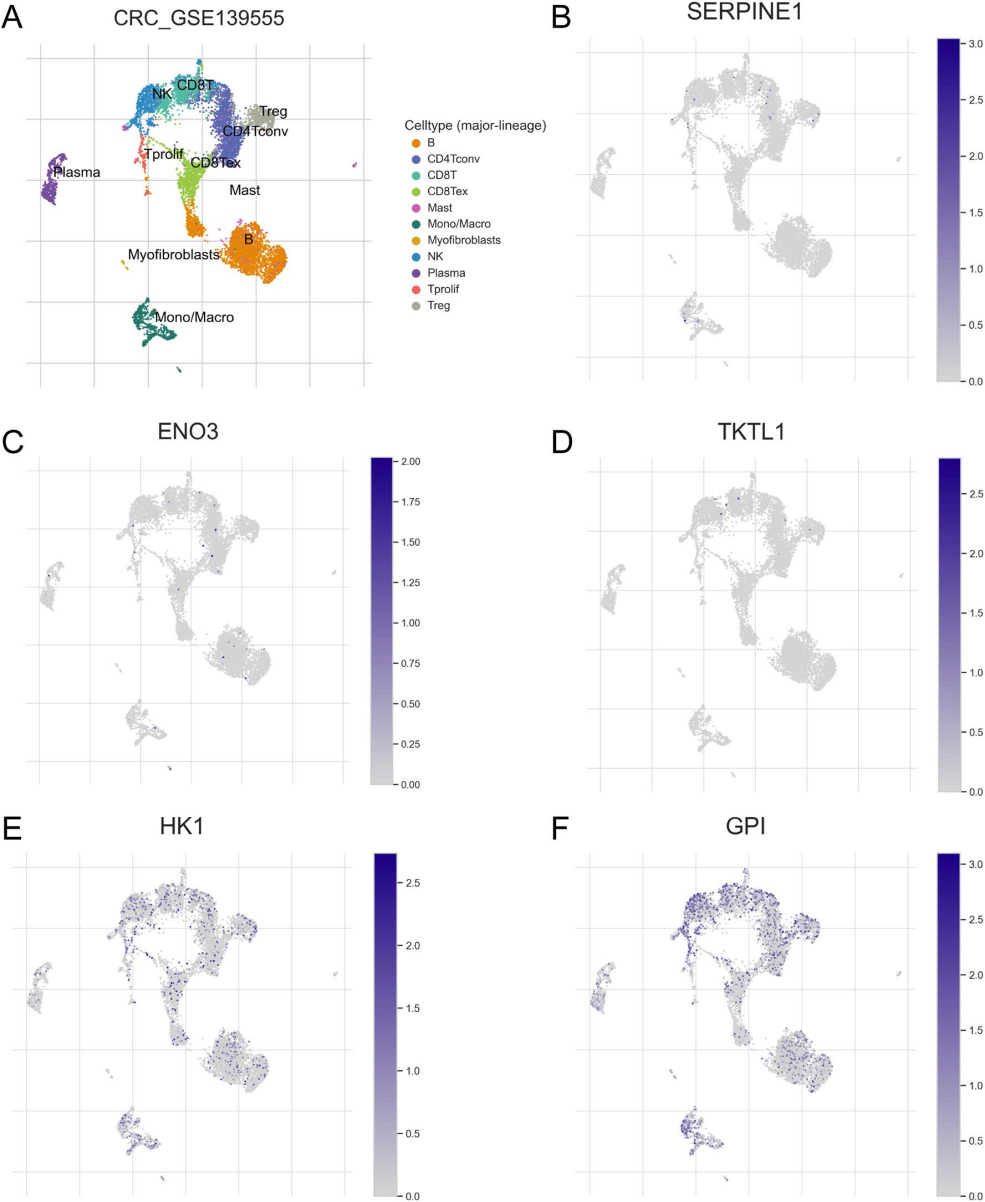
Single-cell RNA-seq analysis of HRGs. (**A**) The distribution of TICs by the UMAP method. The mRNA expression of (**B**) *SERPINE1*, (**C**) *ENO3*, (**D**) *TKTL1*, (**E**) *HK1*, and (**F**) *GPI* in the different TICs.

The different TICs were grouped by the UMAP method (Fig. 6A), and their expression of the signature’s six HRGs is shown in Fig. 6B-F. *ENO3*, *SENPINE1*, and *TKTL1* expression did not vary among TICs (Fig. 6B–D). However, while *GPI* and *HK1* were universally expressed in all TICs, their levels were elevated in NK and CD8^+^T cells (Fig. 6E,F). However, *ALDOB* was not expressed in any TICs.

### HRG expression validation in CRC tissues

**Figure 7:**
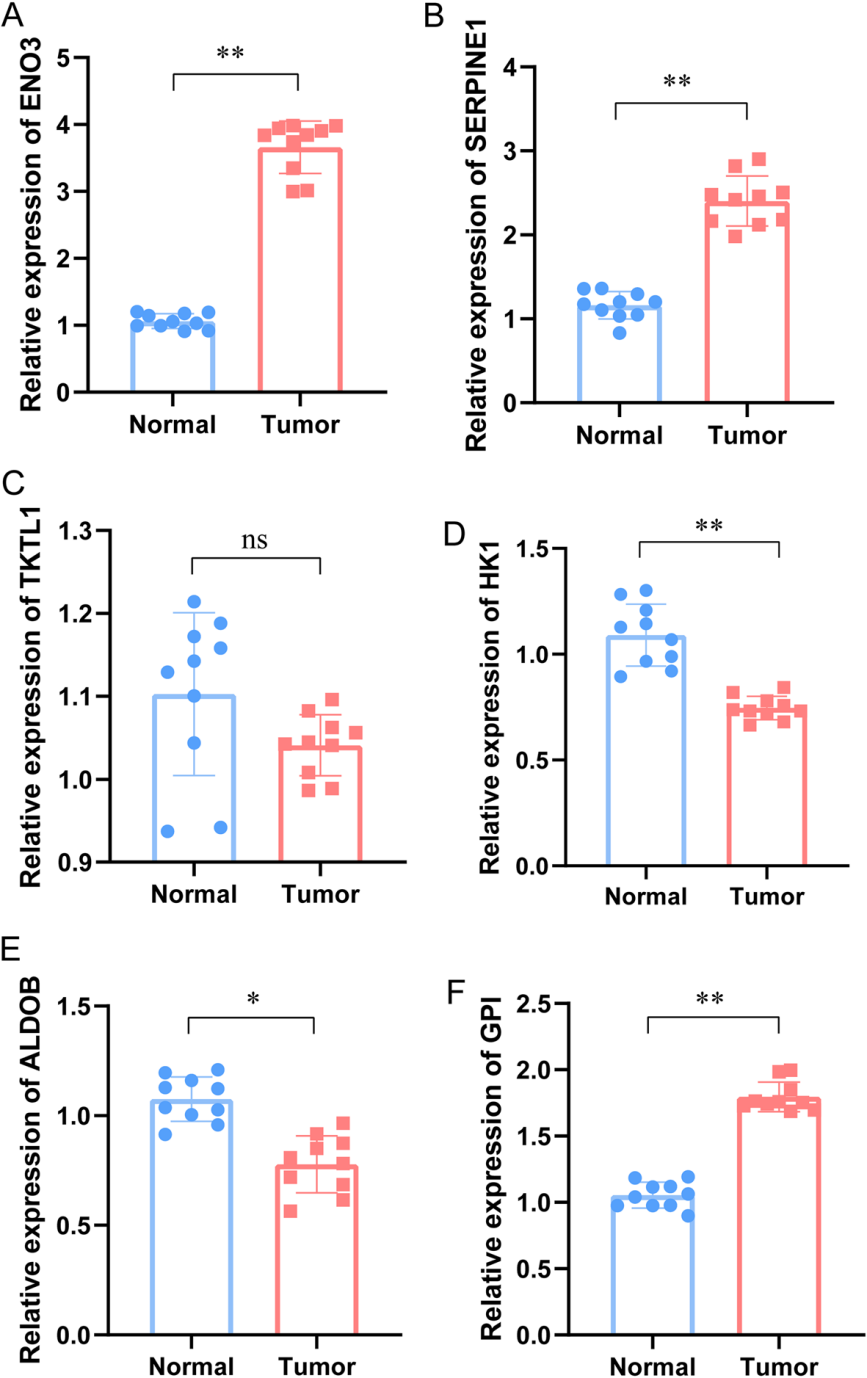
Expression of the signature’s six HRGs in normal and CRC tissues. The mRNA expression of (**A**) *ENO3*, (**B**) *SERPINE1*, (**C**) *TKTL1*, (**D**) *HK1*, (**E)** *ALDOB*, and (**F**) *GPI*. Key: *, *P* < 0.05; **, *P* < 0.01.

We collected ten tumor-normal tissue pairs from patients in Xijing Hospital. After extracting the tissues’ mRNA, RT-PCR analysis showed *ENO3*, *SENPINE1*, and *GPI* upregulation and *HK1* and *ALDOB* downregulation in cancerous compared to adjacent noncancerous tissues (Fig. 7).

## Discussion

In this study, we first studied the mRNA levels of 51 reported HRGs in CRC and normal tissues, finding 12 differentially expressed. To further assess their prognostic value, we constructed a six-gene risk signature via Cox univariate analysis, which we then validated in a GEO dataset. Functional enrichment analysis showed that the DEGs between the low- and high-risk groups were related to several pathways. We examined immune cell infiltration and immune-related genes in the low- and high-risk groups.

This study identified six prognostic HRGs: *ENO3*, *SERPINE1*, *HK1*, *TKTL1*, *ALDOB*, and *GPI*. ENO3 is a major enzyme participating in CRC glycolysis. Low *ENO3* expression has been reported to correlate significantly with prolonged OS in CRC patients. Therefore, *ENO3* might be a promising prognostic biomarker for CRC patients^22^. Besides CRC, ENO3 also functions in pancreatic ductal adenocarcinoma (PDAC). To better predict the OS of PDAC patients, a prognostic nomogram involving ENO3 might contribute to the individualized management of PDAC patients^23^. *ENO3* overexpression results directly from the loss of STK11 function. Moreover, *ENO3* knockdown had a selective anticancer effect in *STK11* mutant lung cells. Therefore, an ENO3-based therapy might be promising for patients with *STK11* mutant lung cancer^24^. ENO3 was enriched in the above HIF-1 pathway.

SERPINE1 is a serine proteinase inhibitor reported to function as a crucial extracellular matrix remodeling regulator. Compelling evidence indicates that SERPINE1 is intimately associated with poor prognoses in diverse cancers. Biochemical analysis showed upregulated SERPINE1 expression in mesenchymal lung cancer. Crosstalk between TGF-β and Yes-associated protein (YAP) signaling pathways might be a precondition for this process^25^. SERPINE1 facilitates neoplastic cell proliferation, migration, and invasion by regulating EMT. High *SERPINE1* expression shortened OS in GAC patients. Therefore, SERPINE1 might function as a novel biomarker and independent prognosticator in GAC patients^26,27^. Hypoxia-mediated ROS upregulated *SERPINE1* expression in MDA-MB-468 breast cancer cells. Therefore, ROS may be an underlying therapeutic strategy for managing breast cancer metastasis^28^.

HK1 participates in distinct biological processes contributing to glycolysis in cancer. HK1 might underlie oncogenes in many tumors^29^. *HK1* overexpression results in pyruvate and lactate overproduction. A detailed study into bladder cancer’s metabolic phenotype might provide novel therapeutic strategies^30^. Previous studies have indicated that enhanced glycolytic activity promotes chemotherapeutic resistance in malignant cancers. However, whether glycolysis affects gemcitabine’s (GEM) curative efficacy in pancreatic cancer (PC) is largely unknown. Under hypoxic conditions, *HK1* expression correlated positively with PC progression, suggesting that upregulated glycolytic activity could facilitate PC progression and increase GEM tolerance^31^.

TKTL1 is an enzyme participating in cancer glycolytic metabolism. TKTL1 was a biomarker for distinguishing CRC patients who might benefit from liver resection from those who might need more aggressive perioperative chemical therapy^32^. TKTL1 participates in aerobic glycolysis in cancer, contributing to basal membrane destruction and cancer metastasis. Besides APO10, TKTL1 is another biomarker underlying epitope detection in monocytes (EDIM) blood tests. By comparing the sensitivity of the EDIM blood test with conventional tumor biomarkers, studies have shown that this novel test might aid the detection and diagnosis of cholangiocellular carcinoma, PC, and CRC^33^.

The aldolase family participates in cancerous metabolism and glycolysis and has three members: ALDOA, ALDOB, and ALDOC. Abnormal aldolase levels are closely associated with several tumor types. ALDOB successfully enhanced glucose uptake and aerobic glycolysis and significantly reduced mitochondrial oxidative phosphorylation in CRC. ALDOB also altered the response of traction force in CRC, which is closely associated with cancer metastasis during hypoxia. Therefore, elevated ALDOB levels could drive hypoxia and stiff substrate to promote aerobic glycolysis in CRC, further deepening our understanding of its role in CRC advancement from a biophysical standpoint^34^.

In hypoxia, GPI maintains glucose metabolism by redirecting the glucose from androgen/androgen receptor (AR)-dependent to hypoxia-induced glycolysis, attenuating chemotherapy efficacy in prostate cancer. However, GPI transcription is suppressed by AR during hypoxia. When GPI was inhibited, therapeutic resistance under hypoxia was reduced, and enzalutamide efficacy was increased^35^. Upregulated glycolysis could improve organ tolerance to hypoxia. Therefore, suppressing glycolysis-related enzyme activity and energy metabolism might be a promising treatment strategy for malignant tumors. One study showed that esculetin could disturb glucose metabolism by binding with GPI, playing an antitumor role^34^.

Altogether, we have shown that hypoxia is closely prognostically associated with CRC and constructed an easy-to-use six HRG signature that is strongly associated with the tumor microenvironment in CRC. Furthermore, we used multiple datasets in order to compensate for the limitations of the individual datasets. This risk signature was an independent risk factor for predicting OS in both TCGA and GEO CRC cohorts. The COX algorithms have limited capabilities and can be supplemented with artificial intelligence algorithms. The DEGs between low and high-risk CRC groups were associated with TICs and immune-related genes. Our study provides a novel six HRG signature for predicting the overall prognosis of CRC patients and provides a robust basis for future studies on the relationship between HRGs and immunity.

## Supplementary Information

Supplementary Table S1.

## Data Availability

The datasets used and analyzed in this study are available from the corresponding author upon reasonable request.The datasets generated and/or analysed during the current study are available in the TCGA and GEO repository, https://portal.gdc.cancer.gov/, https://www.ncbi.nlm.nih.gov/geo/.
