# Supplementary material for: Identification of a hypoxia-related gene prognostic signature in colorectal cancer based on bulk and single-cell RNA-seq
Source: Sci Rep. 2023 Feb 13;13:2503. doi: 10.1038/s41598-023-29718-2 (PMC9925779; doi:10.1038/s41598-023-29718-2)
Supplement: Supplementary file 1 — Supplementary Table S1. [file 41598_2023_29718_MOESM1_ESM.docx]

Table S1 Primers for RT-PCR in the study

| Gene | Primer sequence |
| --- | --- |
| ENO3 | Forward 5’- GGCTGGTTACCCAGACAAGG-3’ |
|  | Reverse 5’- TCGTACTTCCCATTGCGATAGAA -3’ |
| SERPINE1 | Forward 5’- ACCGCAACGTGGTTTTCTCA-3’ |
|  | Reverse 5’- TTGAATCCCATAGCTGCTTGAAT-3’ |
| TKTL1 | Forward 5’- ACAAGCAGTCAGATCCAGAGA-3’ |
|  | Reverse 5’ - TAGCTGGCCCTGTCGAAGTA-3’ |
| HK1 | Forward 5’ - GCTCTCCGATGAAACTCTCATAG-3’ |
|  | Reverse 5’ - GGACCTTACGAATGTTGGCAA-3’ |
| ALDOB | Forward 5’ - GGCAGTTCCGAGAAATCCTCT-3’ |
|  | Reverse 5’ - CTCCTTGGTCTAACTTGATTCCC-3’ |
| GPI | Forward 5’ - CAAGGACCGCTTCAACCACTT-3’ |
|  | Reverse 5’ - CCAGGATGGGTGTGTTTGACC-3’ |
